# Butyric and valeric glycerides blend prevents adverse impacts of coccidiosis challenge in broiler chickens

**DOI:** 10.1016/j.psj.2025.105879

**Published:** 2025-09-22

**Authors:** Sm Mostafizur Rahaman Sumon, Alip Kumar, Di Wu, Shu-Biao Wu, Kosar Gharib-Naseri

**Affiliations:** a School of Environmental and Rural Science, University of New England, NSW 2351, Australia; b Perstorp Animal Nutrition, Singapore

**Keywords:** Coccidiosis, Gut health, Welfare, Organic acid, Broiler

## Abstract

Organic acids are well known for their antibacterial and antifungal effects, but their potential efficacy against coccidian parasites remains underexplored. The aim of the study was to evaluate the effects of a butyric and valeric glyceride blend **(BVg)** in mitigating the adverse impacts of coccidiosis in broiler chickens. A total of 960 mixed-sex d-old Cobb 500 chicks were randomly allocated to five treatments with 12 replicates, each containing 16 birds. The five treatments were: non-challenged control **(NC)**; coccidiosis challenged control **(CC)**; CC+ BVg (**BV**; 500, 500, and 250 g/ton in the starter, grower and finisher phases, respectively); CC + anticoccidial salinomycin, 60 g/t **(AntS)**; and CC + salinomycin + BVg **(ABV)**. Coccidiosis challenge was induced on d9 via oral gavage with *Eimeria* spp. Performance parameters were determined on days 8, 19, 28 and 35. Lesion scoring and ileal digesta sampling was performed on d16 and excreta samples were collected on d20 for oocyst enumeration. One-way ANOVA followed by Tukey’s test to separate means was applied to normally distributed data, while the Kruskal-Wallis test was used for data that did not meet the normality assumption. The results showed that BVg supplementation significantly improved feed conversion ratio **(FCR)** during starter (d0-8), grower (d8-19) and overall study (d0-35) periods compared to the CC group (*P* < 0.05). BVg supplementation also numerically improved flock uniformity and significantly reduced oocyst counts (*P* < 0.05) and duodenal lesions (*P* < 0.05) compared to the CC group. Additionally, BVg inclusion shifted hock burn lesions and tibia length values from the CC group towards the NC and ABV groups, respectively. These findings suggest that BVg may offer a promising nutritional strategy to mitigate the adverse effects of coccidiosis, reducing oocyst loads and gut lesions, while supporting the feed efficiency and hock burn lesions of broiler chickens.

## Introduction

Coccidiosis is a widespread protozoan disease affecting livestock, particularly poultry and cattle, characterized by high morbidity ranging from acute bloody enteritis with high mortality, to subclinical disease. In poultry, coccidia are generally host-specific, with different species infecting specific sections of the intestine (Mesa-Pineda et al., 2021). In chickens, the disease is caused by intracellular protozoa of the phylum Apicomplexa belonging to the genus *Eimeria,* and seven species (viz., *E. tenella, E. necatrix, E. acervulina, E. maxima, E. brunetti, E. mitis, and E. praecox*) are known to infect with varying degrees of pathogenicity and specificity (Lopez-Osorio et al., 2020). *Eimeria* oocysts are highly infectious and relatively resistant to common disinfectants with transmission occurring through ingestion of sporulated oocysts (Jenkins et al., 2019). Once ingested, the parasites invade intestinal epithelial cells during the endogenous (schizogony and gametogony) stages of their life cycle, causing severe mucosal damage (Mesa-Pineda et al., 2021). This results in bloody diarrhea, impaired nutrient absorption, and creates an environment conducive to bacterial proliferation (Allen and Fetterer, 2002). The severity of coccidiosis depends on the number of ingested oocysts and immune status of birds, with young and immunocompromised birds being particularly susceptible (McDougald et al., 2020). Furthermore, mucosal damage facilitates the extravasation of plasma proteins into the intestinal lumen, promoting the proliferation of *Clostridium perfringens*, the main cause of necrotic enteritis in poultry (Prescott et al., 2016). The overall impacts of coccidiosis on poultry production are associated with reduced performance, increased mortality, compromised welfare, and a higher risk of product contamination, posing a major economic challenge (Adhikari et al., 2020). The global annual cost of coccidiosis in chicken production was estimated at approximately £10.36 billion (2016 prices), encompassing both production losses and prophylaxis and treatment costs (Blake et al., 2020). Prophylactic anticoccidial drugs and vaccines are usually used as effective strategies to prevent and control coccidiosis for extended periods of time (Adhikari et al., 2020; Mesa-Pineda et al., 2021). However, several limitations have emerged including: (1) drug resistance from continuous coccidiostat use (Khazandi, 2006); (2) public concerns over drug residues in broiler’s meat; (3) limited production capacity of current vaccine lines (Pastor-Fernandez et al., 2020); (4) reduced growth performance due to intestinal epithelium damage from live vaccines (Stringfellow et al., 2011); and (5) increased cost associated with developing new drugs and vaccines (Nouri, 2023). With some coccidiostats and ionophores now banned in poultry production, coccidiosis has risen pushing the poultry industry to develop nutrition-based alternatives that support more sustainable and antimicrobial-free production (Adhikari et al., 2020). Among potential alternatives, organic acids **(OAs)** have gained significant attention and proven their favourable effects on gut health and bird performance under normal and different disease challenge models (Rathnayake et al., 2021).

The OA compounds are naturally present in animal or plant tissues and are usually found esterified with glycerol to form triglycerides and phosphoglycerides or their derivates (Granstad et al., 2020; Scicutella et al., 2021). Commonly used OAs in poultry production include short-chain fatty acids (**SCFAs**; e.g., formic, acetic, propionic, butyric, and valeric acids), medium-chain fatty acids (**MCFAs**; e.g., caproic, caprylic, capric, and lauric acids) and carboxylic acids bearing hydroxyl groups such as lactic, malic, tartaric, and citric acids (Adhikari et al., 2020; Scicutella et al., 2021; Ebeid and Al-Homidan, 2022; Khan et al., 2022). These compounds primarily exert their effects through antibacterial activity, gut pH reduction, immune system enhancement, gut microbiota modulation, and improvements in gut integrity and nutrient digestibility, all of which collectively enhance poultry production performance (Gadde et al., 2017; Mora et al., 2020; Khan et al., 2022). While the antibacterial and antifungal efficacy of OAs are well established, their effectiveness against coccidian parasites has been rarely investigated. Gut pH reduction induced by OAs has been reported to adversely affect oocysts, leading to less severe intestinal damage (Abbas et al., 2011; Nouri, 2022). *In vitro* studies have shown that butyric acid **(BA)** at 0.8 %, 1 %, and 1.2 % concentrations inhibits oocyst sporulation and damages coccidian oocysts in a dose-dependent manner (Zurisha et al., 2021). Valeric acid **(VA)** compounds, a comparatively newer feed additive, have been less studied with no prior studies on its efficacy against coccidiosis in broiler chickens. However, VA supplementation has shown to improve performance and mitigate the suppressive effects of necrotic enteritis in broiler chickens (Onrust et al., 2018; Hofacre et al., 2020). Notably, blend of OAs has been suggested to have synergistic effects, offering greater benefits compared to individual OAs (Polycarpo et al., 2017; Abdelli et al., 2020). OAs should persist until reaching the lower gut, where many of the anaerobic and facultative pathogens exist. To achieve this, esterification of BA and VA to glycerol enhances their stability and allows them to be activated by intestinal lipase (Bedford and Gong, 2018). However, coccidiosis continues to cause major economic and welfare challenges in poultry, while reliance on anticoccidial drugs and vaccines is limited by resistance, consumer concerns, and cost. Despite the well-documented benefits of BA and VA on growth performance and poultry gut health, their efficacy against coccidiosis in broilers remains underexplored. This study hypothesized that dietary supplementation of butyric and valeric acid glycerides blend **(BVg)** improves broiler performance by mitigating the negative effects of coccidiosis. Additionally, it was hypothesized that combining BVg with the anticoccidial drug salinomycin may exert a synergistic effect, further enhancing the performance and health of the birds. Therefore, this study aimed to evaluate the potential effects of BVg, and its combination with salinomycin on growth performance, oocyst shedding, gut lesions, and welfare parameters including hock burn, footpad dermatitis, litter quality and leg bone morphology in broiler chickens under coccidiosis challenge.

## Materials and methods

### Animal ethics

The experimental procedures applied in the current study were approved by the Animal Ethics Committee of the University of New England, Armidale, NSW 2351, Australia (ARA23-032). The experiment was conducted following the guidelines set for the care and use of laboratory animals for scientific purposes accredited by the Australian Bureau of Animal Health (NHMRC, 2013).

### Housing and management

A total of 960 one-day-old Cobb 500 chicks were sourced from Baiada Hatchery in Tamworth, NSW, Australia. Upon arrival, the chicks were weighed and randomly allocated into 60 pens, each stocked with 16 birds and had a uniform pen body weight (± 20 g/pen) to ensure no significant difference in initial pen weight. Birds were housed in floor pens (1.07 m^2^) with soft wood shavings (approximately 7-8 cm in depth) as bedding materials in an environmentally controlled chicken shed at the Centre for Animal Research and Training (**CART**), University of New England, NSW. Each pen was equipped with a bell feeder and two nipple drinkers, providing ad libitum access to feed and water. The lighting, temperature and humidity of the shed were regulated in accordance with Cobb 500 management guidelines (Cobb500, 2022a). The starting temperature was 33°C and was gradually reduced by 4°C per week to reach the target temperature of 21°C over three weeks, which was then consistently maintained thereafter.

### Experimental design and treatments

The experiment utilized a randomized design with 5 treatment groups, each replicated 12 times with 16 birds per pen. The details of the treatments and experimental design are presented in Table 1. Since the challenge was introduced on d9, birds in the NC and CC pens were treated as a single group (Control) until d8 marking the end of the starter phase. The additive used in the experiment included a blend of butyric and valeric glycerides (Gastrivix™ Avi, Perstorp Waspik BV, Singapore) at 500, 500, and 250 g/ton in the starter, grower and finisher phases, respectively. The basal diet, based on wheat, soybean meal and sorghum, was formulated to meet the nutritional requirements of Cobb 500 broilers (Cobb 500, 2022b), incorporating the nutrient and matrix values of phytase (Axtra PHY GOLD® 10000 FTU/g) at 1500 FTU/kg and xylanase (AXTRA® XB) at 100 g/ton with 3 % energy uplift of main energy source ingredients. Both the feed additive and antibiotic were top-dressed and thoroughly mixed with other ingredients before pelleting. The diets were provided in three phases: starter (d0-8), grower (d8-19), and finisher (d19-35). The nutrient content of feed ingredients was determined prior to feed formulation using near-infrared spectroscopy (AminoNIR®, Evonik Amino Prox, Essen, Germany). Crumbled diets were used during the starter phase, while cold-pelleted diets were used in the grower and finisher phases. In this study, feed was pelleted at 65-70 °C at the CART, University of New England, which is lower than the > 80 °C conditioning temperatures typically used in commercial feed mills. However, pelleting at 65-70 °C reflects practical conditions in smaller-scale or research-based feed production, while helping to preserve heat-sensitive nutrients and maintain pellet durability. Detailed diet composition and nutrient contents for each phase are shown in Table 2.

**Table 1:** Experimental design and treatments.

Treatments^1^	Inclusion rate of Additive/Coccidiostat (%)			Coccidiosis Challenge
	Starter d0-8	Grower d8-19	Finisher d19-35	
NC	0	0	0	No
CC	0	0	0	Challenged
BV	0.05	0.05	0.025	Challenged
AntS	0.05	0.05	0.05	Challenged
ABV	Salinomycin: 0.05 + BVg: 0.05	Salinomycin: 0.05 + BVg: 0.05	Salinomycin: 0.05 + BVg: 0.025	Challenged

1 NC: non-challenged control; CC: challenged control; BV: blend of butyric and valeric glycerides (BVg); AntS: coccidiostat salinomycin; ABV: combination of salinomycin and BVg.

**Table 2:** Composition and nutrient contents of experimental diets.

Ingredients (as fed basis, %)	Starter (d0-8)	Grower (d8-19)	Finisher (d19-35)
Wheat	51.2	45.3	50.7
Soybean meal	29.3	24.9	20.0
Sorghum	12.0	22.2	22.8
Meat and bone meal	2.25	1.50	0.550
Canola oil	1.10	1.45	1.65
Limestone	1.01	1.11	1.12
Dicalcium phosphate	0.414	0.001	0.00
L-lysine HCl	0.390	0.402	0.455
D, l-methionine	0.338	0.319	0.306
L-arginine HCl	0.125	0.150	0.192
L-threonine	0.170	0.150	0.150
UNE TM concentration^1^	0.100	0.100	0.080
UNE Vit concentration^2^	0.080	0.080	0.100
Salt	0.178	0.156	0.143
Choline Chloride	0.058	0.087	0.108
Na bicarb	0.036	0.107	0.100
Phytase^3^	0.015	0.015	0.015
Xylanase	0.010	0.010	0.010
Titanium dioxide	0.000	0.500	0.000
Sand^4^	1.25	1.40	1.43
Total	100.0	100.0	100.0
**Calculated nutrients**^5^			
AME, kcal/kg	2976	3025	3100
Crude p rotein, %	22.9	21.0	18.9
Crude fat, %	3.16	3.49	3.58
Crude fiber, %	2.85	2.71	2.59
Digestible arginine, %	1.37	1.25	1.14
Digestible lysine, %	1.26	1.16	1.08
Digestible methionine, %	0.640	0.597	0.560
Digestible Met + Cys, %	0.94	0.880	0.827
Digestible tryptophan, %	0.259	0.236	0.212
Digestible isoleucine, %	0.817	0.744	0.660
Digestible threonine, %	0.867	0.783	0.710
Digestible valine, %	0.888	0.816	0.731
Calcium	0.960	0.840	0.760
Available phosphorus, %	0.540	0.420	0.381
Sodium, %	0.180	0.180	0.180
Potassium, %	0.941	0.856	0.768
Chloride, %	0.250	0.240	0.240
Linoleic acid (18:2), %	0.947	0.949	1.01
Choline, mg/kg	1750	1750	1750

AME= apparent metabolizable energy.

1 Vitamin premix provided the following per kilogram diet: vitamin A, 12,000,000 IU; vitamin D, 5000,000 IU; vitamin E, 75 mg; vitamin K, 3 mg; cyanocobalamin,0.016 mg; folic acid, 2 mg; riboflavin, 8 mg; pyridoxine, 5 mg; biotin, 0.25 mg; thiamine, 3 mg; nicotinic acid, 55 mg; pantothenic acid, 13 mg and antioxidant ethoxyquin, 50 mg.

2 Mineral premix provided the following per kilogram diet: Cu sulfate, 16 mg; Mn sulfate, 60 mg; Mn oxide, 60 mg; I (iodide), 0.125 mg; Se (selenite), 0.3 mg; Fe sulfate,40 mg; Zn oxide and sulfate, 100 mg.

3 Phytase : Axtra PHY GOLD TPT 10000 FTU/g at 150 g/t.

4 Sand was replaced with the required amount of additive and/or coccidiostat and added to the top.

5 Nutrient contents of major ingredients were measured prior to the feed formulation using near-infrared spectroscopy (NIRS, Evonik AminoProx, Germany).

### Coccidiosis challenge

The coccidiosis challenge was induced following a previously established protocol (Daneshmand et al., 2023). On d9, all birds in the challenged groups were gavaged with one mL of live sporulated *Eimeria* strains containing *E. acervulina* (5000 oocysts), *E. maxima* (5000 oocysts) and *E. brunetti* (2500 oocysts) provided by Eimeria Pty Ltd (Ringwood, VIC, Australia). Birds in the NC group received sterile phosphate buffer saline at 1 mL/bird *per os* as a sham treatment.

### Performance measurements

Pen weights and remaining feed of each pen were recorded at the end of starter (d8), grower (d19), and finisher (d35) phases to calculate the weight gain (**WG**), feed intake (**FI**), and feed conversion ratio (**FCR**). These performance data were used to calculate the performance parameters of entire trial period (d0-35). FI was determined based on the dry matter (**DM**) content of the feed (both feed-in and feed-out). The FCR was also calculated on a DM basis. However, both feed intake and FCR values are presented on an 88 % DM basis (Noblet et al., 2015). The number and weight of dead birds were recorded daily, and these figures were used to correct the FI and FCR. Necropsies were performed to determine the cause of death, and all deceased birds, along with sampled and remaining birds at day 35, were examined to determine sex via visual inspection of the testes.

### Flock uniformity

All individual live broiler chickens were weighed on d35. Flock uniformity was determined by calculating the coefficient of variation (CV%) of live weight of all birds according to the treatment. The CV% was calculated using the following formula.

$$CV\%=\frac{Standard\;deviation\;\left(SD\right)}{Average\;weight}\;\times 100$$

### Sampling and lesion scoring

On d16, four birds were randomly selected from each pen and electrically stunned. The birds were then euthanized by cervical dislocation, followed by dissection. The entire length of the small intestine was removed to assess coccidiosis lesions. Lesion scoring was conducted by two experienced researchers who were blinded to the treatment groups, using a scoring system from 0 to 4 (Johnson and Reid, 1970). Pooled ileal digesta samples from four birds were collected and stored at 4°C until oocyst counting. On day 20, freshly voided excreta droppings (at least five droppings from each pen) were pooled and mixed, and a small amount was transferred to 2 mL Eppendorf tubes and stored at 4°C for oocyst enumeration. The oocyst count was performed within seven days of sample collection.

### *Eimeria* oocyst count

Ileal digesta and excreta sample preparation was performed using the modified McMaster egg counting technique previously described by Sumon et al. (2025). Briefly, about 100 mg of each excreta samples were diluted with 900 μL of saturated salt solution (relative density 1.3) in an Eppendorf tube. After thorough mixing by vortexing, the samples were refrigerated at 4°C for 1 hour to allow the oocysts to float and the debris to settle. Following this, 600 μL of the saturated salt solution was added to the Whitlock chamber (Whitlock universal slides, JA Whitlock & Co., NSW 2122, Australia), and 150 μL of the diluted sample was carefully pipetted from the top layer into the chamber. Oocysts were then counted using a 10× objective lens, and the results were expressed as oocysts per gram of wet excreta, with counts from the chamber multiplied by 100 to account for the dilution factor.

### Litter quality and moisture determination

On d35, litter quality in each pen was assessed through visual inspection using a standard scoring method outlined by Kheravii et al. (2017). The scoring system ranged from 0 to 3, where 0 = dry litter, 1 = slightly caked/moist litter, 2 = moderately caked/moist litter, and 3 = wet litter. Observations were made at four different points within each pen, and the average score was calculated to determine the overall litter score for each pen. On the same day, approximately one kg of litter samples was collected from six specific locations within each pen (near feeders, drinkers, and endpoints) and placed in plastic bags. The collected samples were pooled and weighed both before and after drying in a forced-air oven at 105°C for 24 hours. Moisture content was then calculated according to the method described by Barker et al. (2013).

### Footpad dermatitis and hock burn scoring

On day 35, footpad dermatitis **(FPD)** and hock burn **(HB)** were assessed in all birds from each pen through visual inspection, following the scoring methodology described by Quality (2009). A 5-point scale was used to evaluate the severity of lesions, where a score of 0 indicated no lesions, and 4 represented the most severe macroscopic lesions. The visual scoring was conducted by two experienced personnel who were blinded to the study design and pen arrangements to ensure unbiased evaluations.

### Bone parameters

On d35, one bird from each pen was randomly selected and weighed, and the right drumstick was separated after euthanasia. The drumstick was defleshed by a knife, and the right tibia was collected. They were then air-dried in a fume hood for 48 h and weight was taken. After that, bone length was measured using a digital calliper. Both the vertical and horizontal external diameters of tibia were measured at the breaking point and then averaged (Swiatkiewicz and Arczewska-Wlosek, 2012). Bone breaking strength was determined by three-point flexural test using an Instron Universal Testing System (Model-300 LX-SPL; Instron® Electromechanical & Industrial Products Group, Norwood, MA, USA). Tibia was placed on an adjustable three-point loading system, with a distance among bone supports of 50 mm, and a vertical force was applied at the midpoint by a 2.54 mm fulcrum. The peak breaking strength (i.e., the force which is required to break the bone) was measured in newtons (N).

### Data analysis

All data generated in this study were initially tested for normality prior to statistical analysis. Data that followed a normal distribution were analysed using parametric tests with the Fit model platform in JMP 16.0 (SAS Institute, Cary, NC, USA). When significant treatment effects were identified, the difference between means were separated by the Tukey’s test. Non-normally distributed data including lesion scores, foot pad scores and *Eimeria* oocyst counts were assessed using the non-parametric Kruskal-Wallis test. The pen was considered as experimental unit (*n* = 60), and female percentage (corrected to dead birds) was included as covariate when a significant sex effect was observed. Statistical significance was determined at *P* < 0.05 and the P value at 0.05 < *P* < 0.1 was considered to show a tendency towards significance. In addition, the term ‘shifting’ was used to describe outcomes that exhibited an intermediate or transitional response between treatment groups, where results were not significantly different from either group but suggested a trend toward recovery, aligning more closely with the control group (Sumon et al., 2025). The mathematical model describing the relationship between the response and treatments in a one-way ANOVA was expressed as:

$$Y_{ij}=\mu +\tau_{i}+\varepsilon_{ij}$$

where:

*Yᵢⱼ*: The observed response for the *j*-th observation in the *i* th treatment group.
*μ*: The overall mean across all treatment groups.
*τᵢ*: The effect of the *i* th treatment (factor level) representing the deviation of that treatment group mean from the overall mean.
ϵᵢⱼ: The random error term *i* th treatment level and the *j*-th observation, accounting for unexplained variation in the data not attributed to treatment effects.

## Results

### Performance

**Fig. 1:**
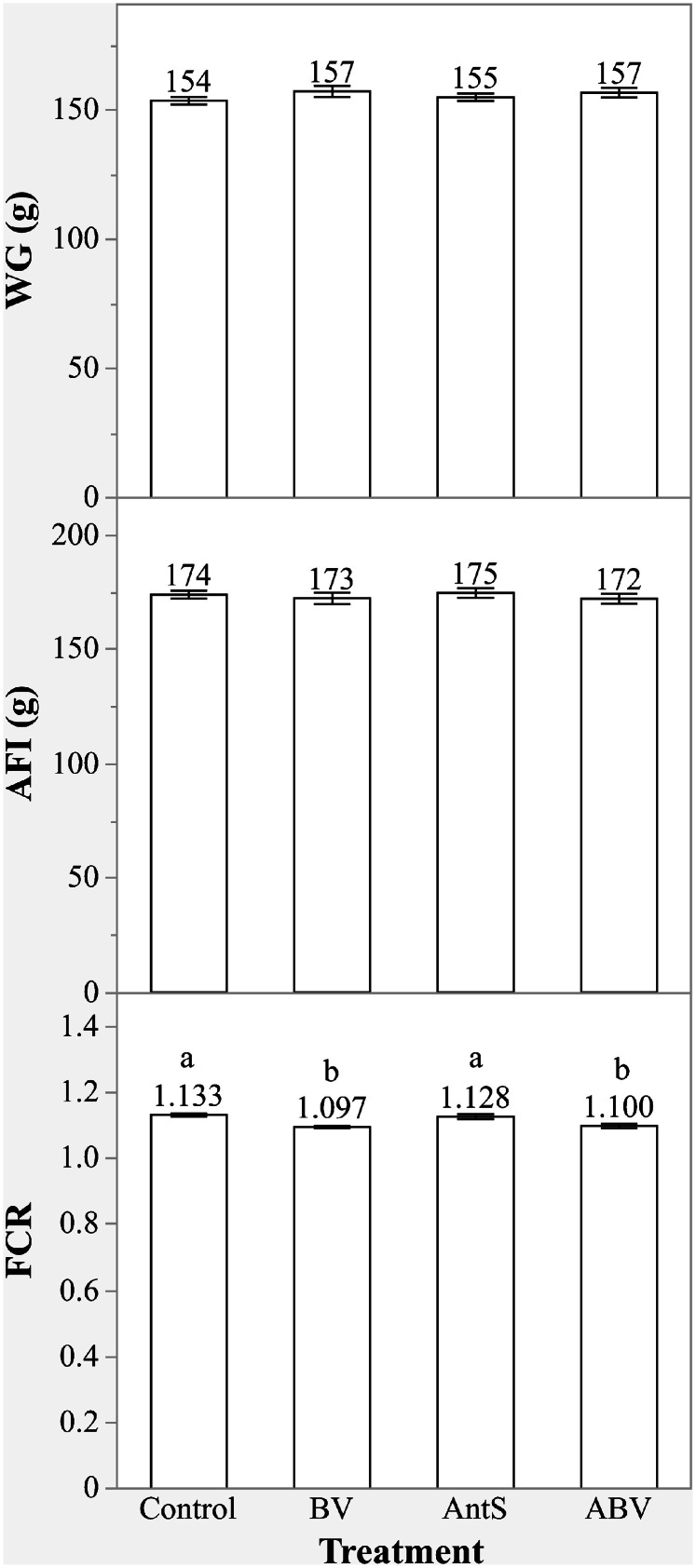
Effect of BVg on growth performance of broilers before inducing coccidiosis challenge (d0-8). ^a-b^Values within a column with different letters differ significantly (*P* < 0.001). Treatment groups: Control= non-challenged birds fed basic diet; BV= non-challenged birds supplemented with BVg (butyric and valeric glyceride blend at 500, 500 and 250 g/ton in starter, grower and finisher phases, respectively); AntS= non-challenged birds supplemented with salinomycin at 60 g/t in all phases; ABV= non-challenged birds supplemented with both the BVg (at 500, 500 and 250 g/ton in starter, grower and finisher phases, respectively) and salinomycin (60 g/t in all phases).

The effects of different treatments on broiler growth performance are shown in Fig. 1 and Table 3. During the starter phase (d0-8), prior to the coccidiosis challenge, BVg, and the combination of BVg and salinomycin (ABV) supplementations significantly improved FCR (3.6 and 3.1 points, respectively) compared to both the control and AntS groups (*P* < 0.05; Fig. 1). However, the ABV supplementation had no benefit over BVg supplementation alone (*P* > 0.05). Neither the additive, the anticoccidial, nor their combination had a significant effect on WG, and FI during this period (*P* > 0.05).

**Table 3:** Effect of BVg on growth performance of broilers under coccidiosis challenge.

Treatment^1^	Grower Phase (d8-19)				Finisher Phase (d19-35)				Overall Study (d0-35)			
	WG (g)	FI^3^ (g)	FCR^3^	Liveability, %	WG (g)	FI (g)	FCR	Liveability, %	WG (g)	FI (g)	FCR	Liveability, %
NC	649^a^	845^a^	1.294^c^	98	1735abc	2675	1.543^ab^	95	2542^a^	3615^a^	1.428^c^	93
CC	462^b^	709^b^	1.539^a^	99	1646^c^	2586	1.573^ab^	95	2264^b^	3440^bc^	1.519^a^	94
BV	476^b^	700^b^	1.467^b^	97	1654^bc^	2569	1.555^ab^	98	2289^b^	3388^c^	1.485^b^	94
AntS	632^a^	835^a^	1.327^c^	97	1747^a^	2651	1.518^b^	97	2532^a^	3609^ab^	1.424^c^	92
ABV	624^a^	833^a^	1.350^c^	98	1736^ab^	2633	1.518^b^	96	2513^a^	3610^ab^	1.431^c^	94
SEM^2^	13	14	0.016	1	22	29	0.010	2	29	38	0.007	2
*P*-value	<0.001	<0.001	<0.001	0.747	0.002	0.117	0.006	0.874	<0.001	<0.001	<0.001	0.983

WG: weight gain; FI: feed intake; FCR: feed conversion ratio.

a-c Values within a column with different letters differ significantly (*P* < 0.05).

1 Treatment groups: NC= Non-challenged control; CC= Challenged control; BV= CC + BVg (butyric and valeric glyceride blend at 500, 500 and 250 g/ton in starter, grower and finisher phases, respectively); AntS= CC + salinomycin at 60 g/t in all phases; ABV= CC + BVg (at 500, 500 and 250 g/ton in starter, grower and finisher phases, respectively) + salinomycin (60 g/t in all phases).

2 SEM: standard error of means.

3 AFI and FCR were based on standardised DM of 88 %.

During the grower phase (d8-19), when the coccidiosis challenge was induced (Table-3), birds in the CC group showed reduced WG (40.5 %) and FI (19.2 %), along with an increased FCR (24.5 points deterioration) compared to the NC group (*P* < 0.05). Birds receiving salinomycin had significantly higher WG (36.8 % and 32.8 %, respectively) and FI (17.8 % and 19.3 %, respectively) and a lower FCR (21.2 and 14.0 points improvement, respectively) than those in the CC and BV groups (*P* < 0.05) but did not differ from the NC group (*P* > 0.05). BVg supplementation in challenged birds significantly improved FCR (7.2 points) compared to the CC group (*P* < 0.05) with no observed effects on WG and FI (*P* > 0.05). However, BVg did not fully mitigate the adverse effects of the coccidiosis challenge on WG, FI and FCR when compared to the NC and AntS groups. The combination of additive and anticoccidial (ABV) significantly improved WG (35.1 %), FI (17.5 %) and FCR (18.9 points) of the challenged birds compared to the CC group (*P* < 0.05). However, no synergistic effect was evident, as ABV supplementation had no additional benefits over salinomycin alone (*P* > 0.05).

In the finisher phase (d19-35), there was no significant difference in WG and FCR between the NC and CC groups (*P* > 0.05). Compared to the CC and BV groups, birds fed salinomycin showed significantly higher (*P* < 0.05) WG (6.1 % and 5.6 %, respectively) and lower FCR (5.5 and 3.7 points improvement, respectively), but did not differ from the NC group (*P* > 0.05). Although WG in the BV group were not different from the NC, CC and ABV groups (*P* > 0.05), the BVg supplementation showed a shift of WG from CC towards ABV group. The FCR in BVg fed birds did not differ from NC, CC, AntS and ABV groups (*P* > 0.05), but there was a shift from CC towards the AntS group. The combined BVg and salinomycin supplementation (ABV) significantly improved WG (5.5 %) and FCR (5.5 points) compared to the CC group (*P* < 0.05), however, did not show complementary effects beyond those observed with BVg or salinomycin alone (*P* > 0.05).

Over the entire experimental period (d0-35), results indicate that the coccidiosis challenge significantly reduced the WG (12.3 %) and FI (5.1 %), while increasing FCR (9.1 points deterioration) in the CC group compared to the NC group (*P* < 0.05). Birds in the AntS group had improved WG (11.8 % and 10.6 % respectively), FI (4.9 % and 5.5 % respectively) and FCR (9.5 and 6.1 points, respectively) compared to the CC and BV groups (*P* < 0.05), with outcomes similar to the NC group (*P* > 0.05). Supplementation of BVg significantly improved FCR (3.4 points) compared to the CC group (*P* < 0.05), although the improvement did not reach the level seen in the NC and AntS groups. The ABV supplementation significantly improved WG (11.0 %) and FCR (8.8 points) compared to the CC group (*P* < 0.05), but did not yield any synergistic effect beyond what was achieved in salinomycin alone (*P* > 0.05).

### Effect on flock uniformity

**Fig. 2:**
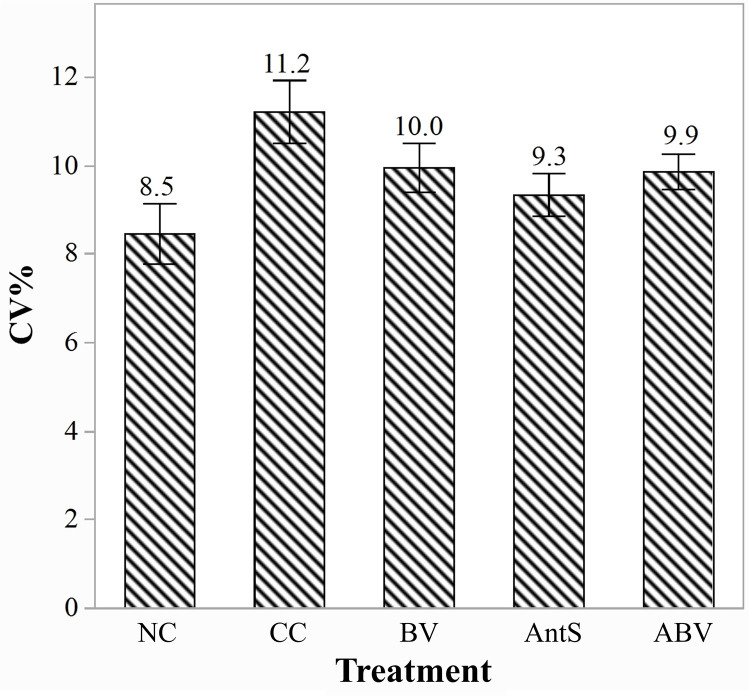
Flock uniformity on d35. Treatment groups: NC= Non-challenged control; CC= Challenged control; BV= CC + BVg (butyric and valeric glyceride blend at 500, 500 and 250 g/ton in starter, grower and finisher phases, respectively); AntS= CC + salinomycin at 60 g/t in all phases; ABV= CC + BVg (at 500, 500 and 250 g/ton in starter, grower and finisher phases, respectively) + salinomycin (60 g/t in all phases). As the flock uniformity was calculated across the whole population of the individual treatments, no replications were possible thus no statistical analysis was performed.

Fig. 2 illustrates the effects of coccidiosis challenge and BVg supplementation on flock uniformity at d35. The CV of body weight across treatment groups followed the order: NC < AntS < ABV < BV < CC. The NC group exhibited the highest flock uniformity with the lowest CV (8.5 %), whereas the CC group showed the lowest uniformity with the highest CV (11.2 %). Birds in the BV, AntS, and ABV groups demonstrated intermediate levels of uniformity, with CVs of 10.0 %, 9.3 %, and 9.9 %, respectively.

### Effect on intestinal lesions

**Fig. 3:**
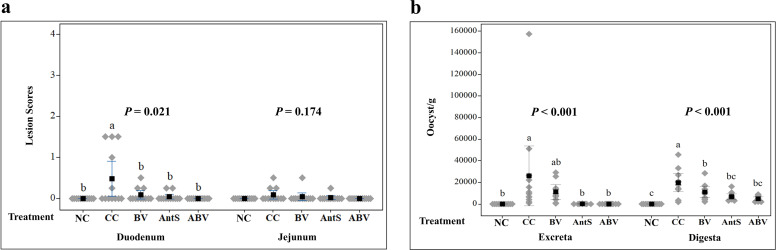
a) Intestinal lesion scores across different treatment groups on d16. b) Oocyst counts in ileal digest (d16) and excreta (d20) samples among different treatment groups. ^a-c^Values within a column with different letters differ significantly (*P* < 0.05). Treatment groups: NC= Non-challenged control; CC= Challenged control; BV= CC + BVg (butyric and valeric glyceride blend at 500, 500 and 250 g/ton in starter, grower and finisher phases, respectively); AntS= CC + salinomycin at 60 g/t in all phases; ABV= CC + BVg (at 500, 500 and 250 g/ton in starter, grower and finisher phases, respectively) + salinomycin (60 g/t in all phases).

Fig. 3a illustrates the effects of the experimental treatments on duodenal and jejunal coccidian lesions, with significant differences observed only in duodenal lesions (*P* = 0.021). Birds in the CC group had significantly higher duodenal lesion scores compared to the NC group (*P* < 0.05). Both salinomycin and BVg supplementation had similar effects (*P* > 0.5) and significantly reduced duodenal lesions compared to the CC groups (*P* < 0.05), with no significant difference from NC group (*P* > 0.05). Birds in the ABV group had significantly lower duodenal lesions compared to the CC group (*P* < 0.05) but was not different from BV and AntS groups (*P* < 0.05), indicating no complementary effect of combining BVg with salinomycin.

### Effect on *Eimeria* oocyst count

Fig. 3b outlines the impact of experimental treatments on the mean *Eimeria* oocyst count in ileal digesta on d16 and in excreta on d20. The experimental treatments had significant effects on both the excreta (*P* < 0.001) and ileal digesta (*P* < 0.001) oocyst counts. Compared to the NC group, birds in the CC group exhibited a significantly higher number of oocysts in both the ileal digesta and excreta samples (*P* < 0.05). Salinomycin fed birds had significantly lower oocyst counts compared to the CC group in both the excreta and digesta samples (*P* < 0.05) but were not different from NC group (*P* < 0.05). BVg supplementation significantly reduced oocyst counts compared to the CC groups in the ileal digesta samples (*P* < 0.05), and its anticoccidial effects did not differ from the AntS group (*P* > 0.05). Although ABV supplementation significantly reduced both the digesta and excreta oocyst counts compared to the CC group (*P* < 0.05), the reductions were not different from BV and AntS groups (*P* > 0.05), suggesting no complementary effect from the combination of BVg and salinomycin.

### Effect on litter quality, hock burn and foot pad lesion

The effects of coccidiosis challenge and supplementations on litter score, moisture percent, hock burn, and foot pad lesions are presented in Table 4. The experimental treatments had a significant effect on hock burn lesions (*P* = 0.003), while no effect was observed on litter score (*P* = 0.060), moisture percent (*P* = 0.078) and foot pad lesions (*P* = 0.294). Birds in the CC group exhibited significantly higher hock burn lesions compared to the NC group. The outcomes of BVg and salinomycin supplementation, whether administered alone or in combination, had the similar effects in reducing hock burn lesions and did not differ significantly from both the NC and CC groups (*P* > 0.05). However, BVg inclusion indicated a shift of hock burn lesions from the CC group towards the NC group.

**Table 4:** Effect of BVg on litter quality, hock burn and footpad lesions of broilers under coccidiosis challenge (d35).

Treatment^1^	Litter Score	Litter Moisture (%)	Hock Burn Lesion Score	Footpad Lesion Score
NC	1.41	24.0	0.18^b^	0.07
CC	1.66	28.1	0.66^a^	0.17
BV	1.53	27.6	0.43^ab^	0.08
AntS	1.38	26.3	0.41^ab^	0.11
ABV	1.53	26.5	0.42^ab^	0.15
SEM^2^	0.08	1.2	0.09	0.12
*P*-value	0.060	0.078	0.003	0.294

a-b Values within a column with different letters differ significantly (*P* < 0.05).

1 Treatment groups: NC= Non-challenged control; CC= Challenged control; BV= CC + BVg (butyric and valeric glyceride blend at 500, 500 and 250 g/ton in starter, grower and finisher phases, respectively); AntS= CC + salinomycin at 60 g/t in all phases; ABV= CC + BVg (at 500, 500 and 250 g/ton in starter, grower and finisher phases, respectively) + salinomycin (60 g/t in all phases).

2 SEM: standard error of means.

### Effect on bone morphology and strength

The dietary treatment effects on tibia morphology and strength of broiler chickens under coccidiosis challenge at d35 are detailed in Table 5. The treatments had significant effects on tibial length (*P* = 0.029) and breaking strength (*P* = 0.047), whereas no significant impact on tibia weight (*P* = 0.169) and diameter (*P* = 0.084). Tibia lengths in the NC, BV, and AntS groups did not differ from CC group (*P* > 0.05). However, birds in the ABV group had significantly longer tibia lengths compared to the CC group (*P* < 0.05). Although tibial length in the BV group was not different from CC group (*P* > 0.05), BVg supplementation showed a shift from CC towards the ABV group, with no significant difference between BV and ABV groups (*P* > 0.05). Tibia breaking strength in the CC group was not significantly different from any other treatment groups (*P* > 0.05). However, birds receiving the combination of BVg and salinomycin (ABV) showed significantly higher breaking strengths compared to the NC groups (*P* < 0.05), but were not different from those in the CC, BV and AntS groups (*P* > 0.05).

**Table 5:** Effect of BVg on tibia morphology and breaking strength of broilers under coccidiosis challenge (d35).

Treatment^1^	Tibia Weight (g)	Tibia Length (mm)	Tibia Diameter (mm)	Breaking Strength (N)
NC	7.54	96.6^ab^	8.94	357.9^b^
CC	7.01	93.4^b^	8.69	386.6^ab^
BV	7.17	94.9^ab^	8.77	408.6^ab^
AntS	7.53	95.4^ab^	8.73	404.4^ab^
ABV	7.65	97.1^a^	8.68	480.8^a^
SEM^2^	0.21	0.85	0.16	28.3
*P*-value	0.169	0.029	0.804	0.047

a-b Values within a column with different letters differ significantly (*P* < 0.05).

1 Treatment groups: NC= Non-challenged control; CC= Challenged Control; BV= CC + BVg (butyric and valeric glyceride blend at 500, 500 and 250 g/ton in starter, grower and finisher phases, respectively); AntS= CC + Salinomycin at 60 g/t in all phases; ABV= CC + BVg (at 500, 500 and 250 g/ton in starter, grower and finisher phases, respectively) + salinomycin (60 g/t in all phases).

2 SEM: standard error of means.

## Discussion

The ban on anticoccidials including ionophores in poultry production, driven by rising drug resistance and consumer concerns, has shifted the research focus towards the control of coccidiosis in broiler chickens by nutritional-based alternatives. Esterified forms of butyric and valeric acids (glycerides) have the ability to maintain the stability of the acids until they reach the hindgut of broiler chickens, where they can function effectively. Hindgut is the primary site of colonization of pathogenic bacteria (Pickard et al., 2017). Acidifying by these glycerides promotes a healthy gut through growth and proliferation of beneficial bacteria and the suppression of the pathogenic ones, nutrient digestion and absorption, anti-inflammatory and immune functions (Khan and Iqbal, 2016; Ebeid and Al-Homidan, 2022). The present study investigated the effects of a combination of butyric and valeric glycerides (BVg) on the performance, gut lesions, oocyst counts and leg bone morphology, as well as the welfare of broiler chickens challenged with coccidiosis. The findings of this study revealed that additive (BVg) supplementation significantly improved FCR during the pre-challenged period (d0-8) and outperformed the CC group during both the challenge period (d8-19) and overall study periods (d0-35). Birds receiving BVg showed significantly lower duodenal lesions and reduced digesta oocyst counts compared to the CC group. Additionally, BVg supplementation reduced hock burn lesions and improved tibial length equating AntS group, with a shift from CC toward the NC and ABV group, respectively. These findings suggest that dietary inclusion of BVg has the ability to prevent adverse impacts of coccidiosis and enhance broiler performance. Based on findings, we conclude that dietary inclusion of BVg enhances broiler performance and welfare such as reduced hock burn lesions by mitigating adverse effects of coccidiosis. In contrast to our anticipation, the combination of BVg with salinomycin did not yield any additional benefit that was observed in birds supplemented with BVg or salinomycin alone. Consequently, the second hypothesis that combining BVg with salinomycin would have synergistic effects and yield greater improvements compared to either BVg or salinomycin alone was rejected.

A successful subclinical coccidiosis challenge was induced as evidenced by lower WG and FI, along with a higher FCR in CC group compared to the NC group during both the challenged (d8-19) and overall (d0-35) study periods. It is well-documented that both the clinical and subclinical coccidiosis are associated with decreased WG, FI and poor FCR in broiler chickens (Wang et al., 2019; Mustafa et al., 2021; Daneshmand et al., 2023; Nouri, 2023). The parasites invade enterocytes and cause severe damage to intestinal epithelium. This was evidenced by the significantly higher oocyst counts and intestinal lesions observed in the CC group of this study, leading to impaired nutrient digestion, absorption, and ultimately reduced growth performance in broiler chickens. In this study, dietary supplementation of BVg significantly improved FCR under coccidiosis challenge particularly during the peak challenge and overall study periods. Notably, the improvement in FCR observed during the pre-challenge period highlights the potential of BVg to improve gut microenvironment, promote nutrient utilization and sustain performance even under impending coccidiosis challenge. Consistent with our findings, Mustafa et al. (2021) reported that supplementation with OAs (a blend of encapsulated butyrate and MCFAs) improved overall broiler performance, both with and without coccidiosis challenge. Similarly, Nouri (2023) demonstrated that encapsulated OAs enhanced broiler performance, carcass traits, oocyst reduction rates, serum cholesterol, globulin, total protein levels, serum and excreta calcium and phosphorus concentrations, jejunal histomorphometry, *mucin-2* gene expression, and modulated intestinal microbial populations, including *Lactobacillus, Escherichia coli* and coliforms under coccidiosis challenge. Additionally, Zurisha et al. (2021) reported that butyric acid supplementation improved performance and reduced oocyst counts and lesion scores in broiler chickens challenged with coccidiosis. In this study, BVg supplementation significantly decreased ileal digesta oocyst counts and duodenal lesion scores, indicating improved gut health and supporting its effectiveness in enhancing performance. This improvement can be attributed to enhanced gut development, improved nutrient digestibility, and modulated gut microflora, inflammation and immunity by OAs, as previously reported (Mora et al., 2020; Khan et al., 2022). In addition, BVg supplementation resulted in an increase in tibial length, statistically similar to AntS which showed shifted results from CC towards ABV group, which is consistent with improved minerals utilization and bone growth. The overall tendency of challenged birds towards exhibiting higher tibial breaking strength than NC birds; with ABV birds showing significantly higher than NC, may be a reflection of the day 35 sample collection and the post-challenge recovery/catch-up mineralization, particularly under the ABV regimen. Whereas in NC the faster growth may have outpaced mineral deposition and reduced mechanical loading, yielding relatively under-mineralized cortices at the same age. These findings warrant further investigation, particularly into mineral utilization, cortical bone quality, and mechanical loading during post-challenge recovery. OAs reduce intestinal pH which promotes availability, digestion and absorption of calcium and phosphorus for chickens (Boling et al., 2000; Nezhad et al., 2011; Pearlin et al., 2020). In addition, butyrate, a key component of BVg, serves as an essential energy source for enterocytes, supports gut mucosal health, and promotes the proliferation and differentiation of epithelial cells, thereby contributing to better nutrient absorption (Canani et al., 2011). On the other hand, valeric acid glycerides have been reported to increase intestinal villi height, resulting in an increased surface area capable of more nutrient absorption and digestion, and to elevate GLP-2 cells in the intestine, which stimulates intestinal growth (Onrust et al., 2018). The improved performance of birds under coccidiosis challenge in this study could be associated with the synergistic and/or complementary effect of butyric and valeric glycerides on gut health. However, in the finisher phase (d19-35), no significant differences in the growth performance were observed between NC and CC groups, possibly reflecting the recovery of the birds from the challenge due to the self-limiting nature of coccidian infection and boosting of performance in the CC groups (Mesa-Pineda et al., 2021). Furthermore, combining BVg with salinomycin did not provide additional benefits beyond those observed with BVg or salinomycin alone, indicating a lack of synergy between these two additives under the tested conditions. It suggests that their mechanisms of action may not complement each other.

*Eimeria* infestation causes severe damage to the intestinal epithelium and increases the faecal oocyst shedding, which facilitates reinfection (McDougald et al., 2020). In this study, coccidiosis challenge significantly increased oocyst counts and duodenal lesions in CC group, while BVg supplementation significantly reduced ileal oocyst counts and duodenal lesion. This reduction in oocyst counts might be attributed to the acidification of the gut environment by the dissociated form of butyric and valeric acids, which creates an unfavourable environment for oocysts (Abbas et al., 2011; Nouri, 2022). Another plausible explanation of anticoccidial effect might be the stimulation of avian host defence peptides **(HDPs)**, as butyrate is known to induce HDPs expression in humans and livestock (Sunkara et al., 2011; Wu et al., 2020). *β*-defensin-1, for example, has been shown to reduce oocyst counts by 2-3 times compared to *Eimeria*-challenged control groups (Mahmoud et al., 2023). HDPs have broad-spectrum antimicrobial activity against bacteria, protozoa, enveloped virus, and fungi, primarily through direct binding and lysis of microbial membranes (Magana et al., 2020; Mookherjee et al., 2020). Similar to our findings, previous work has shown that butyric acid administration inhibits oocyst sporulation and damages coccidian oocysts *in vitro*, while improving lesion scores and oocyst counts *in vivo* (Zurisha et al., 2021). Beyond the direct effect on coccidian cells, improved gut immunity, integrity and barrier function by BVg supplementation could prevent the invasion of epithelial cells by parasites, resulting in reduced intestinal lesions (Sumon et al., 2025). However, the reduced oocyst count observed in the BVg group in this study might be attributed to a lowering of gut pH by the dissociated forms of BA and VA glycerides in the gut, and the stimulation of avian HDPs, particularly β-defensin-1, which collectively exert detrimental effects on *Eimeria* oocysts.

Litter quality, foot pad dermatitis (FPD) and hock burn (HB) lesions are well stablished indicators of housing conditions and bird welfare (Haslam et al., 2007). In this study, neither the additive, anticoccidial treatment nor the coccidia challenge significantly affected litter quality or FPD lesions compared to the CC group. Although not statistically significant, FPD lesions in the CC group were 2.5 times higher than in the NC group (0.17 vs. 0.07). On the other hand, birds in the CC group exhibited significantly higher HB lesions compared to the NC group. Previous studies have reported a positive correlation between HB and FPD lesions (Meluzzi et al., 2008). BVg supplementation showed no difference in HB lesions from both CC and NC groups although CC significantly differ from NC. This is an indication of a HB lesion shift from CC towards the NC group led by the BVg treatment. Litter quality, a key factor affecting foot pad health (Shepherd and Fairchild, 2010), is affected by environmental factors (e.g., housing and litter materials), diet (e.g., dietary electrolytes and viscous grains), and enteric pathogens (e.g., *Eimeria* spp. and *C. perfringens*) (Dunlop et al., 2016; Swiatkiewicz et al., 2017). Despite expectations that coccidiosis challenge could deteriorate litter quality due to diarrhea, the mild challenge model used here did not result in severe enteritis. Furthermore, litter quality was assessed on d35, when birds were in the recovery phase due to the self-limiting nature of the disease, which likely contributed to the lack of significant differences in litter quality and FPD lesions. Although, HB, FPD lesions and litter quality are influenced by each other, the observed shift in HB lesions suggests a beneficial effect of BVg on chicken welfare under coccidiosis conditions.

## Conclusion

Dietary supplementation with BVg significantly improved FCR in birds challenged with coccidiosis, particularly during peak challenge period and over the entire study period. Notably, improvement in FCR was also observed during the starter phase, prior to coccidiosis challenge, suggesting BVg’s potential to support growth performance and health even under impending coccidia challenge. Furthermore, dietary inclusion of BVg reduced oocyst counts and duodenal lesions, suggesting a protective effect against coccidian parasites and reducing epithelial damage, which may have contributed to improved nutrient utilisation, ultimately enhancing overall performance and welfare. However, this study was limited to a single BVg inclusion level, which restricts conclusions on dose-response relationships and optimal supplementation strategies, and it did not incorporate detailed gut health measurements or evaluate potential synergistic effects with other feed additives. Further research should aim to optimize BVg inclusion levels, explore its interaction with other additives and investigate the underlying mechanisms contributing to its benefits to further elucidate its role in mitigating coccidiosis and enhancing broiler productivity.

## CRediT authorship contribution statement

**Sm Mostafizur Rahaman Sumon:** Writing – original draft, Investigation, Conceptualization. **Alip Kumar:** Writing – review & editing, Supervision, Investigation. **Di Wu:** Funding acquisition, Writing – review & editing. **Shu-Biao Wu:** Writing – review & editing, Supervision. **Kosar Gharib-Naseri:** Writing – review & editing, Supervision, Data curation.

## Disclosures

We declare that we have no financial and personal relationships with other people or organizations that can inappropriately influence our work, there is no professional or other personal interest of any nature or kind in any product, service and/or company that could be construed as influencing the content of this paper.
